# Reproductive Performance of Mouse Oocyte after *In Vivo* Exposure of The Ovary to Continuous Wave Ultrasound

**Published:** 2012-12-17

**Authors:** Nahid Nasiri, Ahmad VosoughTaqi Dizaj, Poopak Eftekhari-Yazdi, Mohammad Reza Akhond

**Affiliations:** 1 Department of Embryology, Reproductive Biomedicine Research Center, Royan Institute for Reproductive Biomedicine, ACECR, Tehran, Iran; 2 Department of Reproductive Imaging, Reproductive Biomedicine Research Center, Royan Institute for Reproductive Biomedicine, ACECR, Tehran, Iran; 3 Department of Statistics, Mathematical Science and Computer Faculty, Shahid Chamran University, Ahvaz, Iran

**Keywords:** Parthenogenesis, Ultrasound, Fertilization Rate, Mouse Oocyte, Blastocyst

## Abstract

**Background:**

There is a lack of studies regarding the effects of ultrasound (US) and replication of its exposure on pre-implantation events in mammals. Thus, this study assesses the reproductive performance of mouse oocytes that have been obtained from ovaries irradiated with US waves versus non-irradiated ovaries. Also comparision of their parthenogenesis, ovulation, fertilization, and pre-implantation development rates.

**Materials and Methods:**

In this experimental study, we divided extracted ovaries into three experimental groups that received the same dosage, but different replicates of radiation for each group. Results were compared with the control and sham groups. Continuous wave (CW) US, at a spatial average intensity of 355 mW/cm^2^ and a frequency of 3.28 MHz, was administered for 5 minutes to the ovaries at an interval between pregnant mare serum gonadotropin (PMSG) and human chorionic gonadotropin (hCG) injections. Statistical analysis was performed using the ANOVA test and the level of significance was determined to be 0.05.

**Results:**

Data collection was based on microscopic visualization. According to the obtained results, metaphase II (MII) oocyte numbers and the percentage of blastocysts significantly reduced in the USexposed groups versus the unexposed groups. Fertilization rate was comparable between groups while parthenogenesis was significantly higher in the US-exposed groups compared to the unexposed groups.

**Conclusion:**

Structural damage to cells, intracellular organelles and proteins, as well as changes in signaling pathways induced by US may be reasons for some of the observed adverse effects in groups that have received more US exposure.

## Introduction

In the past several decades, the use of diagnostic ultrasound (US) for follicle development assessment and oocyte acquisition has increased. US has two potentially destructive effects (thermal and mechanical) on biological systems (1 , 2 ). With regard to frequent and repeated application of US in obstetrics and gynecology practices, there is growing concern about the theoretical possibility of deleterious effects on oocyte function and subsequent embryo development. Because of the insufficient amount of studies to confirm the safe use of US, there is a necessity for additional research. Some accomplished animal and human studies have implicated that US causes deleterious effects on oocytes and subsequent embryo development. For example, the study by Heyner et al. (3 ) has shown that US can significantly reduce ovulation rates in mice. In addition, an induced adverse effect of meiosis resumption of oocytes has been noted by Testartet al. (4 ). Miyoshi et al. (5 ) observed that US can induce parthenogenic activation of pig oocytes. Despite the completion of several studies about the effect of US on fertilization and preimplantation development, there is no evidence about its adverse effects as well as the effect of exposure repetition on such variables.The goal of this study is to examine the effects of a continuous wave (CW) US at a spatial average intensity of 355 mW/cm^2^ (at diagnostic range) and the effect of simultaneously repeated radiation on four important reproductive events (the number of metaphase II (MII) oocytes, parthenogenesis, fertilization, and preimplantation development) of mouse oocytes and resulting zygotes.

## Materials and Methods

This experimental study was initially approved by the Ethical and Scientific Committee of Royan Institute.

### Animals

A total of 202 mature 6-8 week old female NMRI mice (Pasteur Experimental Animal Supply, Karaj, Iran) were used in this study. The mice were housed in metal cages and kept in a room with controlled lighting (12 hours light: 12 hours dark cycle) and temperature (22-24ºC) with ad libitum access to commercial pellet and water.

### Exposure system and calibration

**Fig 1:**
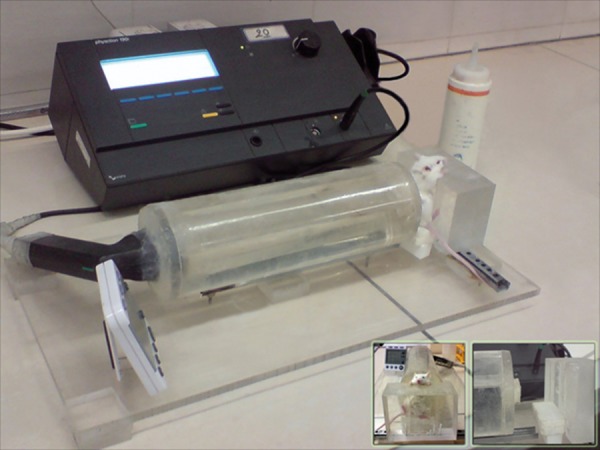
The exposure system and mouse fulcrum. The chamber of system was filled with degassed water. The cyclic section from the center of the left lid was removed, and the US probe was positioned centrally and perpendicular to the chamber. The anesthetized mouse was placed on the cubic piece of yonolit at the opposite end of the chamber. The mouse’s paws were placed on top of the fulcrum. The ventral face of the fulcrum was concave and the mouse’s abdomen was placed inside it. A sliding metaliferous rail was positioned under the mouse fulcrum. The axial distance between the mouse ovary and probe was 25.5 cm. At the right end of the chamber there was a cylindrical plastic prominence with a 1 cm diameter to ensure accurate targeting of the mouse ovary.

The exposure of the mice ovaries to CW US was carried out with a 4 cm diameter transducer (Phyaction 190i, Germany), at a frequency of 3.28 ± 0.18 MHz and spatial average intensity of 355 mW/cm^2^. Figure 1 shows the exposure system and mouse fulcrum made of perspex, which is a good acoustic absorber that minimizes wave reflections from the chamber walls and prevents a second crossing of the waves from the ovaries. The chamber of system was filled with distilled degassed water. The cyclic section from the center of the left lid was removed and the US probe was positioned centrally and perpendicular to the chamber. The anesthetized mouse was placed on the cubic piece of yonolit at the opposite end of the chamber. The mouse’s paws were placed on top of the fulcrum, which consisted of a cubic piece of perspex (a transparent thermoplastic) that minimized wave reflection toward the ovary after passage of the wave through the body. Its ventral face was concave and the mouse’s abdomen was placed inside it. The space between mouse’s abdominal skin and the fulcrum was filled with Sonostat diagnostic couplant gel. A sliding metaliferous rail was positioned under the mouse fulcrum in order to facilitate movement toward the left and right. The mouse seat was also movable, allowing for ease of exposure to both ovaries. The axial distance between the mouse ovary and probe was equal to the last axial maximum point (25.5 cm), thus the chamber length was 25.5 cm. At the right end of the chamber (the location of the mouse-chamber contact) there was a cylindrical plastic prominence with a 1 cm diameter to ensure accurate targeting of the mouse ovary, which was of a very small thickness to minimize the standing wave (Fig1). Intensity measurement was carried out employing a calibrate needle hydrophone (Precision Acoustics, 1 mm needle, England). Radiation was carried out at an interval between human chorionic gonadotropin (hCG) and pregnant mare serum gonadotropin (PMSG) injections.

### Ovulation stimulation

Female adult mice (6-8 weeks) were super-ovulated with intraperitoneal (i.p.) injections of 5 IU of PMSG (Organon, Holland) at 8 pm followed 48 hours later by 5 IU of hCG (Organon, Holland). Radiation to mice ovaries was accomplished in the time interval between PMSG and hCG injections.

### Ovarian radiation

In this study there were seven groups: control (n=30) that only underwent hyper-stimulation; three experimental groups of mice (US1, US2, US3) that underwent one exposure on day 1 of the PMSG injection (US1), two exposures (first and third days before injection of hCG; US2), and three exposures (first, second, and third days at an interval between the PMSG and hCG injections; US3); and three sham groups who underwent treatments similar to the experimental groups except that the US device was turned off during exposure time.

Mice scheduled to receive US were anesthetized with 1 mg ketamine and 1 mg xylazine (Alfasan-WOC-RDEN, Holland) diluted in 4.6 ml distilled water, and administered at a dose of 0.2 ml/30 g/mouse. In order to determine the ovary’s location, we dissected 12 mice in the pilot study. The location of the ovarian fat pad was noted through the dorsal wall, an area approximately 2×2 cm^2^ on the back of the mouse (skin area above the ovary) was determined, and the hair in this section was shaved. Approximately 0.5 ml of an aqueous gel was used as a coupling agent and placed onto the skin’s surface. The mouse was then placed vertically on a holder in the exposure system (Fig 1) and the prominence of the right end of the chamber was connected to the skin covered with gel. Next, the device was turned on and ovaries were exposed to the US for five minutes.

In order to evaluate the temperature rise induced by US during radiation, we used a thermocouple to measure temperature changes. A fine T type (copper/ constantan wire) thermocouple (22 μm, Hayoung, NX4-03) probe with 0.01ºC sensitivity and a temperature monitoring speed of <0.001 seconds was calibrated with a thermocycler (Eppendorf, Germany) and placed on the skin area above the ovary. in contact at the interface between the coupling gel and the skin. Temperatures were recorded at one minute intervals for six minutes.

### Oocyte collection

At 16 hours post-hCG injection, super-ovulated mice were killed by cervical dislocation; the oviducts were removed and transferred to T6 medium with 4% bovine serum albumin (BSA; Sigma, A-3311, USA). Ovulated oocytes surrounded by cumulus cells (COC) were released after dissection of the ampulla. COC were washed three times with fresh T6 medium to partial isolation of granulosa cells around the oocytes. Parthenogenic oocytes (in the form of 2-cell embryos) were counted by microscopic visualization before transferring other oocytes into the fertilization medium.

### Sperm preparation

Old NMRI male mice (3-5 months) were housed singly for at least five days before sperm collection. After killing each mouse, both epididymes were removed and placed in T6 medium with 15% BSA. Sperm were collected from the epididymes after cutting 4-5 times with surgical scissors.

### in vitro fertilization and embryo culture

About ten collected COCs were placed in each 150 μl fertilization drop (T6 + 15% BSA) in which the sperm had already been incubated for at least 15 minutes in order to induce capacitation. Dishes were placed in an incubator at 5% O_2_ and 5% CO_2_, balanced in 90% N2 and maintained at 37ºC for 5 hours. Subsequently, eggs were washed to clear excess sperm and groups of ten embryos were randomly selected and placed in 20 μl drops of T6 medium with 4% BSA under mineral oil. Embryos were cultured over 96 hours to the blastocyst stage at 37ºC in a humidified atmosphere of 5% O_2_ in air.

### Statistical analysis

First, we assessed for normality of the distribution of continuous variables by the Kolmogorov- Smirnov test. The difference between two means was tested by one-way ANOVA if the variance was uniform; otherwise they were tested by the Kruskal-Wallis test. Pair-wise comparison was accomplished by Turkey’s procedure. P<0.05 was considered significant.

## Results

### In vivo thermometry

In vivo temperature rises induced by the US were evaluated in 12 female mice.The final temperature increase on the skin above the ovary after 5 minutes of radiation was 1.171 ± 0.13ºC (mean ± SE).

### MII and parthenogenic oocytes

In this study, 2387MII oocytes from 202 female mice were collected 16 hours after hCG injection. The results of the MII and parthenogenic oocyte count as well as those for the remaining variables are shown in table 1. The number of MII oocytes in groups under US exposure was significantly lower than in the control and sham groups (p<0.05). Of the 3191collected oocytes, 313(9.8%) had activated parthenogenically. There was a statistically significant increase in the number of parthenogenic oocytes in the US3 group (p<0.05).

### Fertilization and blastocyst formation rates

Of the 2387 MII oocytes, 1955 (81.9%) embryos were formed. Table 1 shows the results of fertilization rate measurement as well as blastocyst formation rate. Fertilization rate did not differ significantly between US-exposed and unexposed groups. Blastocyt formation was significantly reduced in the US-exposed groups (p<0.05).

## Discussion

Sonography, a technique which uses US waves to detect many events inside the body, is known as one of the safest techniques as confirmed by studies (6 ). However, further application of this technique requires additional research and evaluation. US waves belong to the category of mechanical waves, thus environmental material is needed for wave diffusion. When matter (i.e. tissue) is exposed to US, a part of the US energy is absorbed by the matter and converted to heat (7 ). One of the two biological effects of US is the thermal effect. In this study, we have determined the maximum increase in temperature after six minutes of radiation to be 1.171 ± 0.13ºC. Since temperature increases of less than 1.5ºC are considered biologically nondestructive (8 ), the observed effects of US in our study can be related to the mechanical effects of US.

US may induce pores on the oocyte plasma membrane, entrance of Ca^2+^ from surrounding granulose cells into the immature oocyte in radiated follicles, and finally lead to a parthenogenic activation of the oocyte (9 ). So, due to parthenogenetically activation of oocytes, it is expected that access to MII oocytes is available before injection of hCG or, in other words, even in the absence of LH induction. In the present study, only US3 group showed a significant increase in the number of parthenogenic oocytes. Because this group had the most radiation exposure, it was likely that immature oocytes in this group matured by US exposure. The oocytes were activated parthenogenically because they were radiated more frequently (three repeats). The number of MII oocytes declined in US-exposed groups when compared with the control and sham groups. Perhaps US might be able to influence early maturation of oocytes (before LH induction), while not affecting the number of oocytes that reach the MII phase. According the Heyner et al. study, a reduction in the rate of MII oocytes could be related to significant temperature elevations (<1.5ºC) in the system during US exposure (3 ). This was not applicable in our study. The actual explanation has yet to be determined and requires further investigation.

The results obtained from *in vitro* fertilization of collected oocytes in the seven groups showed no significant differences in the fertilization rates observed between the US-exposed and unexposed groups. A non-significant increase in fertilization rate in US2 group was seen.

Ca^2+^ plays an important role in the fertilization process. The results of past studies have shown that Ca^2+^ remains in the oocyte for several hours after fertilization (10 , 11 ), and that long-term reception of Ca^2+^ is necessary for successful fertilization (12 , 13 ). However in our study, US exposure to immature oocytes following Ca^2+^ exchange was carried out just hours before fertilization, thus it can be presumed that the US did not influence fertilization.

**Table 1:** Comparison of outcomes between study groups


Groups	Mice (n)	MII oocytes retrieved (n)	Parthenogenic oocytes	Fertilization rate (%)	Blastocysts (n)

**Control**	30	10.45 ± 0.74^a, b^	1.66 ± 0.19	82.58 ± 4.54	33.81 ± 4.86^q^
**US1**	27	13.17 ± 0.89^b^	1.29 ± 0.20	79.64 ± 4.12	31.70 ± 4.05
**US2**	30	10.24 ± 0.57^c, d^	1.73 ± 0.22	90.21 ± 4.36	25.05 ± 4.80^b, c^
**US3**	28	12.53 ± 0.90^d^	1.34 ± 0.21	82.45 ± 3.98	36.63 ± 4.35^c^
**Sham1**	30	10.08 ± 0.59^e^	1.85 ± 0.17^a, b^	80.14 ± 4.82	19.95 ± 3.96^a, b^
**Sham2**	29	11.77 ± 0.91	1.30 ± 0.20^b^	75.98 ± 4.22	27.41 ± 3.77^e^
**Sham3**	28	13.93 ± 0.89^a,c, e^	1.34 ± 0.17^a^	84 ± 4.15	38.50 ± 4.64b^b, d, e^


Values are mean ± SE. In each column, groups with at least one similar letter, have significant difference (p<0.05). US; Mice in these experimental groups were irradiated one (US1), two (US2), and three (US3) times by US. Sham: Treatments in these groups were similar to the experiment groups except that the US device was turned off during exposure times.

The effect of US on blastocyst formation as a final step of pre-implantation development was also studied. Little is known about the agents affecting embryo development to the blastocyst stage after the exposure of follicles by US. The normal development of mouse embryos requires the presence of enough good quality oocytes (14 , 15 ) and cell proliferation (16 ),in addition to other factors. In mice, 5 or 6 rounds of cell division are required for blastocysts to form (16 ). It has been observed that at least 24 hours following cellular radiation by US, their proliferation power level has reduced by 22%(17 ). In addition, free radicals generated by US radiation can reduce the proliferation power of cells for several future generations (18 ). Oocyte quality influences early embryonic survival and developmental capability;this competence is acquired during the oocyte maturation period (15 ). There are evidences that US can induce some events which affect oocyte quality. For example, it has been observed that an US can cause destruction and accumulation of intracellular organelles (19 ). Another observed effect is an elevation of the apoptosis rate in cells (17 ). An US may be able to change the cell destination by the activation of a specific protein or signaling pathway (20 ). It is clear that the signaling pathway plays an important role in the determination of cell fate.An US might be capable of changing protein function (according to the frequency resonance hypothesis) via a change in the threedimensional structure of the protein or through decomposing the multi-molecular complex of the protein (20 ).

As we previously noted, under US exposure conditions, the concentration of intracellular Ca^2+^ increased, which might activates the enzymatic pathway and inhibites the energy producing system of the cell (15 ). Finally, it has been observed that oocytes, zygotes, and the number of normal resultant embryos significantly affect the production of blastocysts *in vitro* (14 ). In this study, a significant reduction in blastocyst formation in the US exposure groups was expected due to the reduced numbers of MII oocytes in these groups.

## Conclusion

As most related studies have suggested, it seems that the use of US waves in the field of obstetrics and gynecology does not lead to any side effects. Because the adverse effects observed in our study belonged to the groups that received the most frequent radiation, we propose that the use of sonography techniques in the diagnostic range and with the minimum amount of repetition is safer. This study did not have any molecular evaluation. For an exact assessment of the observed events, further studies in the fields of genetic and molecular biology are required.
